# CD45 phosphatase is crucial for human and murine acute myeloid leukemia maintenance through its localization in lipid rafts

**DOI:** 10.18632/oncotarget.11622

**Published:** 2016-08-25

**Authors:** Laetitia Saint-Paul, Chi-Hung Nguyen, Anne Buffière, Jean-Paul Pais de Barros, Arlette Hammann, Corinne Landras-Guetta, Rodolphe Filomenko, Marie-Lorraine Chrétien, Pauline Johnson, Jean-Noël Bastie, Laurent Delva, Ronan Quéré

**Affiliations:** ^1^ Inserm UMR866, Université Bourgogne-Franche-Comté, Dijon, France; ^2^ LipSTIC Labex, Dijon, France; ^3^ Institut Curie, PSL Research University, UMR9187–U1196, CNRS-Institut Curie, Inserm, Centre Universitaire, Orsay, France; ^4^ Plateforme de lipidomique, Université Bourgogne-Franche-Comté, Dijon, France; ^5^ Plateforme de cytométrie, Université Bourgogne-Franche-Comté, Dijon, France; ^6^ INRA, UMR1324, Dijon, France; ^7^ Hôpital Universitaire François-Mitterrand, Service d'Hématologie Clinique, Dijon, France; ^8^ Department of Microbiology and Immunology, Life Sciences Institute, University of British Columbia, Vancouver, British Columbia, Canada

**Keywords:** acute myeloid leukemia, CD45 phosphatase, lipid rafts, hematopoietic cells, oncogenic transformation

## Abstract

CD45 is a pan-leukocyte protein with tyrosine phosphatase activity involved in the regulation of signal transduction in hematopoiesis. Exploiting CD45 KO mice and lentiviral shRNA, we prove the crucial role that CD45 plays in acute myeloid leukemia (AML) development and maintenance. We discovered that CD45 does not colocalize with lipid rafts on murine and human non-transformed hematopoietic cells. Using a mouse model, we proved that CD45 positioning within lipid rafts is modified during their oncogenic transformation to AML. CD45 colocalized with lipid rafts on AML cells, which contributes to elevated GM-CSF signal intensity involved in proliferation of leukemic cells. We furthermore proved that the GM-CSF/Lyn/Stat3 pathway that contributes to growth of leukemic cells could be profoundly affected, by using a new plasma membrane disrupting agent, which rapidly delocalized CD45 away from lipid rafts. We provide evidence that this mechanism is also effective on human primary AML samples and xenograft transplantation. In conclusion, this study highlights the emerging evidence of the involvement of lipid rafts in oncogenic development of AML and the targeting of CD45 positioning among lipid rafts as a new strategy in the treatment of AML.

## INTRODUCTION

CD45 is a member of the protein tyrosine phosphatase (PTP) family expressed on all hematopoietic cells, except mature erythrocytes and platelets. CD45 dephosphorylates Src kinases involved in the regulation of several cytokine-receptor signal transduction pathways, such as the granulocyte/macrophage colony-stimulating factor (GM-CSF). GM-CSF receptor activation and downstream signaling is a key driver in the pathogenesis of acute myeloid leukemia (AML). GM-CSF receptor is expressed in more than 80% of AML patients' blast cells [1-3]. AML shows evidence of autocrine production of the cytokine and overexpression of its receptor [4, 5]. Constitutive activation of GM-CSF survival pathway has also been reported in AML [6]. GM-CSF induces anti-apoptotic signals in acute myeloid leukemia (AML) [7]. Stat transcription factors are essential for GM-CSF-regulated processes and constitutive activation of this pathway results in enhanced transcription of anti-apoptotic, survival and cell cycle progression genes, which contribute to the pathogenesis of various myeloid leukemias [8]. Different strategies have also been employed to inhibit the GM-CSF receptor on AML blasts [9, 10].

In myeloid cells, CD45 regulates Lyn (Src) on its tyrosine inhibitory site (Y507), and phosphorylation at this site correlates with inefficient signaling through the GM-CSF receptor [11-13]. The localization of CD45 phosphatase within or outside plasma-membrane lipid rafts is crucial for dephosphorylation of Src family kinases, and while this regulation has been thoroughly studied for normal lymphocyte maturation and activation in the immune response [14-16], very few studies have investigated myeloid cells, and even fewer in the context of leukemogenesis.

Using a well-recognized mouse model, human primary samples and a xenograft model, we discovered that a new plasma membrane binding agent belonging to the *Pyrido [4,3-b]quinoxaline* (*PyQ*) family blocks the GM-CSF-dependent proliferation of AML leukemic cells. This molecular mechanism is unique because CD45, which is mainly found within plasma-membrane lipid rafts on AML cells, is rapidly delocalized after treatment and therefore the effect of its phosphatase activity is subsequently profoundly affected. Interestingly, given the fact that CD45 is not localized within lipid rafts of non-transformed murine and human hematopoietic cells, lower toxicity is observed in normal primitive hematopoietic cells.

## RESULTS

### A new chemical compound affects growth of leukemic cells, but does not impact normal hematopoiesis

**Figure 1:**
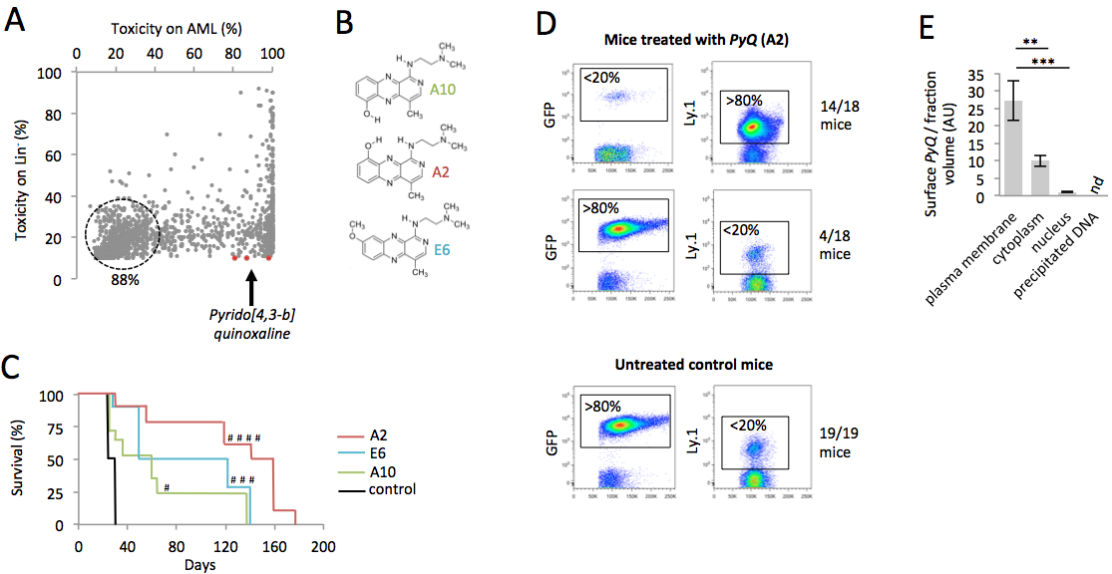
A chemical compound binding to plasma membrane exhibits toxicity on AML cells **A.** Scatter plot showing the toxicity of more than 7,400 indole chemical compounds (10ng/mL) after 18 hours of *in vitro* culture on HOXA9-MEIS1 and Lin^−^cells. **B.** Chemical structures of the *PyQ* compounds A2, E6 and A10. **C.** Kaplan-Meier survival curves of HOXA9-MEIS1 mice treated with A2, E6 or A10 (3mg/Kg), compared with control groups. Control; *n* = 19 mice, A10; *n* = 17 mice, E6; *n* = 10 mice, A2; *n* = 18 mice from two different donors. **D.** Quantification by flow cytometry of the leukemic cells (GFP^+^) and hematopoietic cells (Ly.1) in peripheral blood, 21 days after the transplantation. Mice were treated with compound A2; *n* = 18. Untreated control mice; *n* = 19. **E.** Localization of *PyQ* (A2) by HPLC chromatography in different subcellular compartments of THP1 cells showing important binding of *PyQ* to plasma membrane, *n* = 3 biological samples. Mean ± SEM. nd, not detected, **, *P* < 0.01; ***, *P* < 0.001; measured by Student's unpaired *t* test. ^#^, *P* < 0.1; ^###^, *P* < 0.001; ^####^, *P* < 0.0001; measured by the Mantel Haenszel logrank test, compared with control group.

Homeobox genes and the cofactor MEIS1 have been directly implicated in hematological malignancies [17-19]. We therefore used bone marrow (BM) transplantation experiments and performed retroviral transduction of lineage-negative (Lin^−^) cells for coexpression of human HOXA9 and MEIS1 genes, to induce rapid development of AML, ∼2 months after transplantation. In order to identify new drugs for the treatment of AML, we analyzed the toxicity of more than 7,400 indole compounds on HOXA9-MEIS1-transformed cells *in vitro*. We based our broad compound selection on high specificity for AML cells, with little effect on Lin^−^ (Figure 1A). We identified three compounds (A2, E6 and A10; Figure 1B), belonging to the *Pyrido [4,3-b]quinoxaline* family. We then tested the effect of these compounds *in vivo* to evaluate their ability to block the development of leukemia. AML cells (5×10^4^ GFP^+^ cells) were transplanted in competition with Lin^−^ hematopoietic cells (5×10^4^ congenic Ly.1) into the tail vein of lethally irradiated recipients. Survival analyses showed that mice treated with *PyQ* survived significantly longer than untreated control mice, and it turned out that A2 was the best compound (Figure 1C). When AML growth was monitored *via* peripheral blood (PB) analysis, three weeks post-transplant, we observed that the untreated control mice had rapidly developed AML ( > 80% of GFP^+^ leukemic cells in PB), while mice treated with *PyQ* displayed a smaller number of leukemic cells ( < 20%), and significantly reconstituted hematopoiesis with healthy hematopoietic cells ( > 80%) (Figure 1D). Low toxicity was furthermore detected on primitive hematopoietic stem cells and progenitors in BM when the compound was injected *in vivo* (Supplementary Figure S1).

### CD45 hematopoietic cells are more sensitive to *Pyrido [4,3-b]quinoxaline*, which target plasma membrane

**Figure 2:**
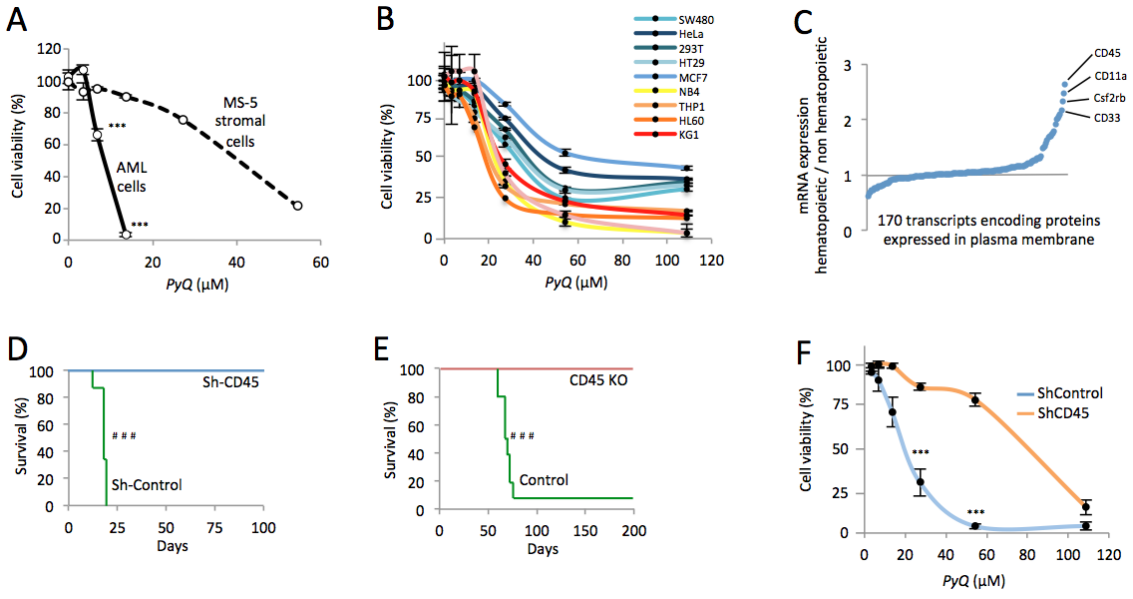
CD45 expression is essential for AML and hematopoietic cells expressing CD45 are more sensitive to *Pyrido [4,3-b]quinoxaline* **A.** HOXA9-MEIS1 cells were grown on MS-5 stromal feeder cells *in vitro*, then treated with *PyQ*. Cell viabilities were measured at different concentrations and data were normalized to untreated control. **B.** Human hematopoietic cell lines were more sensitive to *PyQ*, compared with non-hematopoietic human cell lines, *n* = 4 biological replicates. **C.** Expression of > 170 mRNA encoding proteins known to be localized in plasma membrane, in hematopoietic cell lines (mean values from NB4, HL60, THP1, KG1 and U937), compared with non-hematopoietic cells (mean values from HeLa, SW480, HT29 and MCF7). Data were obtained by computational analysis of the GSE57083 microarray study. **D.** Using shRNA lentivirus, CD45 was knocked down in AML cells and transplanted into lethally irradiated recipient mice. Kaplan-Meier survival curves showing that CD45 was critical for the maintenance of HOXA9-MEIS1 cells, Sh-Control; *n* = 8 mice, Sh-CD45; *n* = 8 mice. **E.** Deficiency in CD45 expression completely prevented CD45 KO primitive hematopoietic cells from leukemic transformation. Kaplan-Meier survival curves, Control; *n* = 10 mice, CD45 KO; *n* = 10 mice. **F.** THP1 cells were transduced with shRNA lentiviral particles (Sh-CD45 or Sh-Control) and treated with different concentrations of *PyQ* for 4 days. Sh-CD45 THP1 cells had lost their sensitivity to *PyQ*, compared with the control line, *n* = 3 biological replicates. Mean ± SEM. ***, *P* < 0.001; measured by Student's unpaired *t* test. ^###^, *P* < 0.001; measured by the Mantel Haenszel logrank test.

We next wanted to understand clearly the intracellular effect induced by this compound. By chromatography performed on different subcellular compartments (Figure 1E), the compound was not detectable in precipitated DNA and no effect was observed on cell cycle activity (Supplementary Figure S2) rejecting the hypothesis that *PyQ* could block replication by intercalating DNA. We furthermore excluded the possibility that *PyQ* could be an inhibitor of kinases (Supplementary Figure S3). In contrast, we interestingly pointed out that *PyQ* interacted strongly with the plasma membrane, with low diffusion into the nucleus (Figure 1E). We confirmed the interaction between *PyQ* and artificially made membranes (Supplementary Figure S4). We hypothesized that *PyQ*, by binding the plasma membrane, might affect the function of intramembranous proteins and their downstream signaling. When we treated HOXA9-MEIS1 cells cultured on MS-5-stromal cells, we surprisingly observed that *PyQ* had a far stronger effect on leukemic cells than on stromal feeder cells (Figure 2A), ant it turned out that human hematopoietic cell lines were more sensitive than non-hematopoietic cells (Figure 2B). We therefore analyzed cell surface proteins that were specifically found expressed by hematopoietic cells (Figure 2C). The most expressed, CD45, is a pan-leukocyte protein with tyrosine phosphatase activity involved in the regulation of several cytokine receptors that control cell growth and proliferation. CD45 is important for the homing and engraftment of leukemic cells [20]. Inhibition of CD45 expression by shRNA lentivirus (Supplementary Figure S5A) prevented AML cells from causing leukemia (Figure 2D and Supplementary Figure S5B), which clearly demonstrates that CD45 expression is essential for the maintenance of AML cells. The deficiency in CD45 expression (CD45KO cells) completely prevented primitive hematopoietic cells from leukemia transformation, demonstrating that CD45 is critical for the development of AML (Figure 2E and Supplementary Figure S5C). Using shRNA lentiviral transduction, we generated CD45 knocked down THP1 cells (Supplementary Figure S6) that had lost their sensitivity to *PyQ* and confirmed that CD45 was required for the inhibitory effect on leukemic cells (Figure 2F).

### CD45 is differently organized on the cell surface of non-transformed hematopoietic cells compared to leukemia-transformed cells

**Figure 3:**
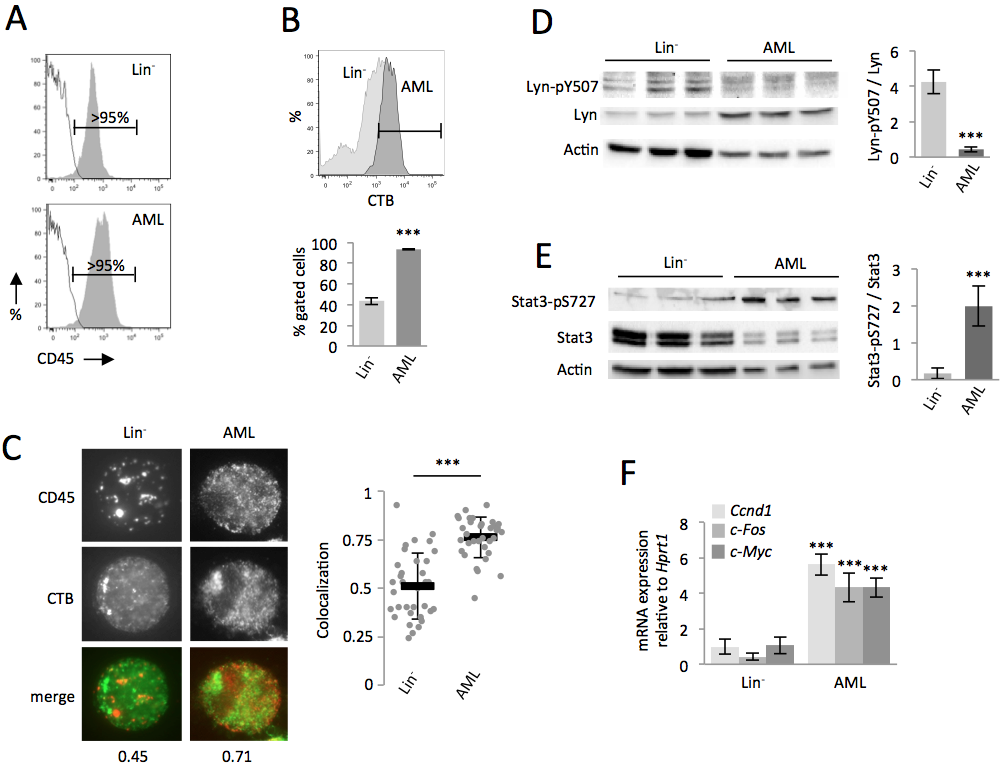
Compared with the non-transformed hematopoietic cells, CD45 colocalized within lipid rafts in AML cells, which enhanced the Lyn/Stat3 pathway **A.** Both Lin^−^ and AML cells expressed high levels of CD45. **B.** Compared with Lin^−^ cells, AML cells are enriched in lipid rafts, as assessed by flow cytometry, with staining with the cholera toxin subunit B (CTB). **C.** Data showing that CD45 colocalized within lipid rafts (CTB) on AML, but not on Lin^−^ cells. Examples of single cell immunostaining were shown on the left panel. Colocalization scores between CD45 and CTB were reported bellow the immunostaining. Colocalization means were calculated (*n* > 30 cells) and represented on the right panel. **D.** On AML cells, CD45 phosphatase localization in lipid rafts increased its activity, which was assessed by decreased phosphorylation of Lyn (Y507), shown by WB, *n* = 3 biological replicates. **E.** The Stat3 pathway is highly activated in AML cells compared with Lin^−^ cells, as assessed by the increased phosphorylation (S727) of Stat3, shown by WB, *n* = 3 biological replicates. **F.** RT-qPCR showing increased transcription of specific Stat3-target genes, *n* = 3 biological replicates. Mean ± SEM. ***, *P* < 0.001; measured by Student's unpaired *t* test.

We next investigated whether the non-transformed cells were less sensitive than AML cells to *PyQ*. The proportion of CD45 could not explain this difference since expression levels were similar (Figure 3A). Lipid rafts are cholesterol- and glycosphingolipid-enriched patches located in the plasma membrane. They are a key component of the signal transduction pathway and contribute to signal intensity modulation in normal hematopoiesis [21-23]. CD45 is associated with lipid raft microdomains, and though the dynamic regulation of CD45 inside or outside lipid rafts has been well documented for lymphocyte activation in the immune response [14], its significance in leukemia is still unclear. Compared with Lin^−^ cells, we observed that AML cells were enriched in lipid rafts (Figure 3B) and therefore investigated the positioning of CD45 within lipid rafts by fluorescence microscopy. CD45 colocalized preferentially within lipid rafts on AML cells, but not on the non-transformed primitive murine hematopoietic cells (Figure 3C). CD45 was not furthermore colocalized within lipid rafts on different populations of hematopoietic stem cells (HSC) and progenitor cells purified from Lin^−^ cells (Supplementary Figure S7). CD45 is a protein tyrosine phosphatase that, when situated within lipid rafts, reduces phosphorylation of the inhibitory site (Y507) of Lyn [24]. This kinase belongs to the Src family, and is activated when dephosphorylated on this site. In AML cells, CD45 was predominantly localized within lipid rafts, the cells consequently displayed relevant tyrosine dephosphorylation at Y507 of Lyn (Figure 3D), leading to an enhancement of its activity in AML cells [25]. Lyn regulates GM-CSF signaling [11, 12], a key pathway implicated in myeloproliferative disorders and leukemogenesis by increasing the proliferative capacity of cells and extending their lifespan [26]. We confirmed the strong activation of the GM-CSF pathway in AML cells, as assessed by an improved phosphorylation of the downstream Stat3 transcription factor (Figure 3E) and enhanced transcription of the Stat3 specific target genes, *c-Myc*, *c-Fos* and *Ccnd1* (Figure 3F), known to be involved in cell cycling and proliferation of leukemic cells [8].

In conclusion, we showed that the localization of CD45 on the cell surface of hematopoietic cells changed during their oncogenic transformation and the Lyn/Stat3 pathway was consequently more activated in AML cells, compared to the non-transformed hematopoietic cells.

### AML cells expressing high levels of CD45 are more leukemogenic and sensitive to *Pyrido [4,3-b]quinoxaline*

**Figure 4:**
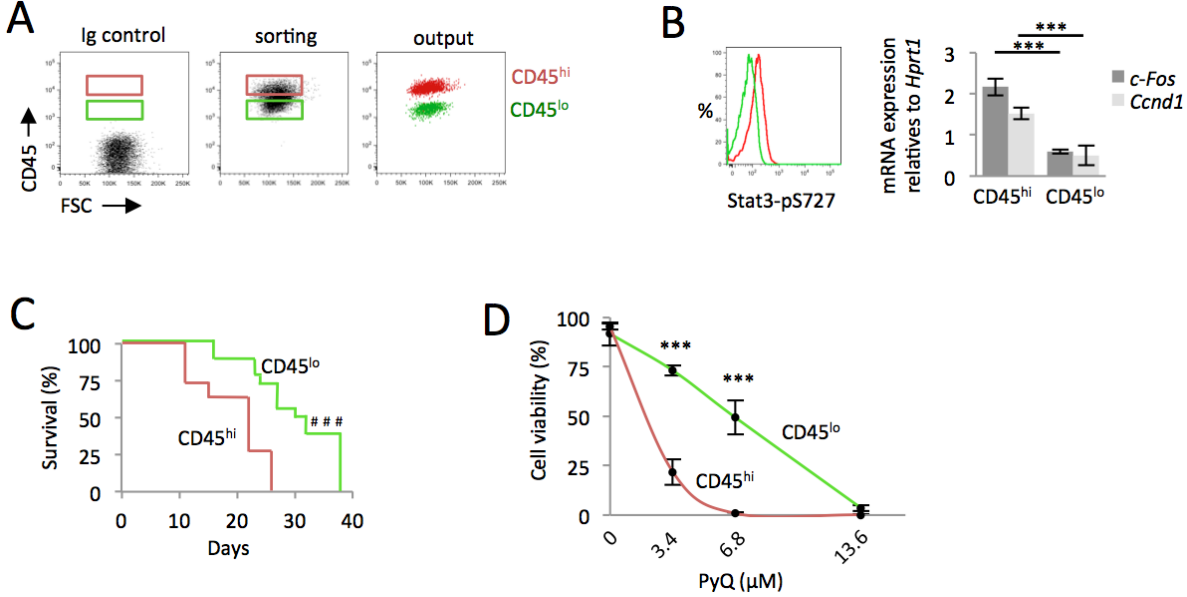
AML cells expressing high level of CD45 are more leukemogenic and sensitive to *Pyrido [4,3-b]quinoxaline* CD45^hi^ cells contained more leukemogenic cell. **A.** HOXA9-MEIS1 cells were stained with anti-CD45 antibody and sorted according to their high or low level of CD45 (CD45^hi^ and CD45^lo^). **B.** Quantification of phospho-Stat3 (S727) by flow cytometry and real-time qPCR of Stat3 target genes indicated that the GM-CSF pathway was up-regulated in CD45^hi^ cells. **C.** CD45^hi^ and CD45^lo^ AML cells analyzed for their ability to induce AML following their intra-femoral injection into recipient mice. Kaplan-Meier survival curves, *n* = 16 mice for each group, showing that CD45^hi^ cells contained more leukemogenic cells. **D.** CD45^hi^ and CD45^lo^ cells were treated with various concentrations of *PyQ* for 18h. Cell viability indicated that CD45^hi^ cells were more sensitive to *PyQ*, *n* = 3 biological replicates. Mean ± SEM. ***, *P* < 0.001; measured by Student's unpaired *t* test. ^###^, *P* < 0.001; measured by the Mantel Haenszel logrank test.

From AML cells, we purified CD45 cells expressing either high or low levels of CD45 phosphatase (*i.e.* CD45^hi^ or CD45^lo^, Figure 4A). Downstream GM-CSF signaling was highly activated on CD45^hi^ cells, as assessed by increased phosphorylation of Stat3 (S727), as well as increased activation of Stat3 target genes (Figure 4B). Through a transplantation study, CD45^hi^ cells were found more leukemogenic (Figure 4C). Cells expressing higher levels of CD45 phosphatase were found to be more sensitive to *PyQ*, further confirming the mechanism by which *PyQ* targets CD45 (Figure 4D).

### Delocalization of CD45 phosphatase away from lipid rafts inactivates the Lyn/Stat3 pathway on AML cells

**Figure 5:**
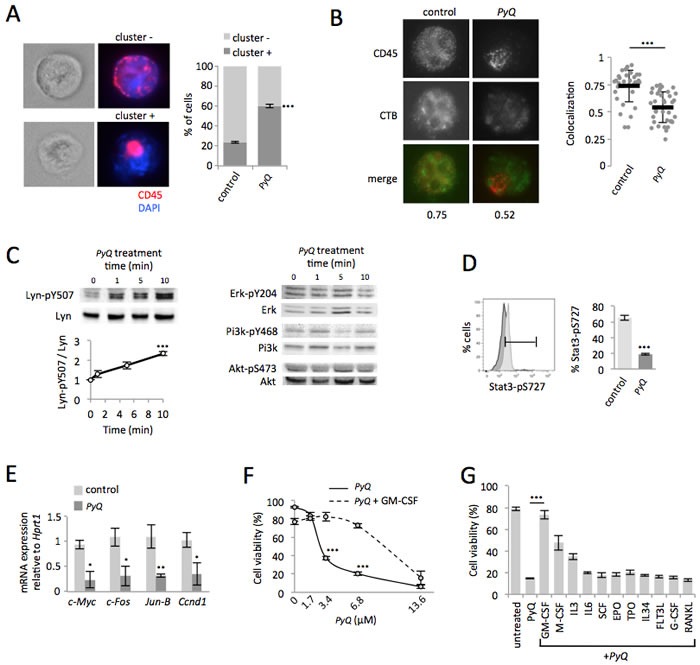
Positioning of CD45 within lipid rafts is crucial for the Lyn/Stat3 proliferation pathway in AML cells **A.** Quantification of CD45 clustering induced by *PyQ* (3.4μM) for 10min on HOXA9-MEIS1 cells. The CD45 was either dispersed on the cell surface (cluster -) or clustered (cluster +). Examples of immunostaining are shown (magnification ×63) and statistics (*n* > 20 cells). **B.** Lipid rafts were stained with cholera toxin subunit B (CTB). For untreated AML cells (control) the CD45 colocalized within lipid rafts. When AML cells were treated with *PyQ* (3.4μM) for 10 min, the CD45 was no longer colocalized with lipid rafts. Examples of single cell immunostaining were shown on the left panel. Colocalization scores between CD45 and CTB were reported bellow the immunostaining. Colocalization means were calculated (*n* > 30 cells) and represented on the right panel. **C.** HOXA9-MEIS1 cells were treated with *PyQ* (3.4μM) for different times. WB and quantification indicated phosphorylation of the negative regulatory site of Lyn (Y507), *n* = 3 biological replicates and WB for Erk, Pi3k and Akt. **D.** As assessed by flow cytometry, phosphorylation of Stat3 (S727) was found reduced 40 min after a treatment with *PyQ*. **E.** As observed by RT-qPCR, specific Stat3 target genes were furthermore inactivated 2 hours after a treatment with *PyQ* (3.4μM), *n* = 3 biological. **F.** Viability of AML cells treated for 18h with various concentrations of *PyQ*, containing or not GM-CSF (25ng/ml), *n* = 3 biological replicates. **G.** Viability of AML cells treated with *PyQ* alone or with several cytokines (25ng/mL). Mean ± SEM. *, *P* < 0.1; **, *P* < 0.01; ***, *P* < 0.001; measured by Student's unpaired *t* test.

We postulated that *PyQ*, by binding to the plasma membrane, could disrupt the organization of intramembranous CD45 at the cell surface. Indeed, when AML cells were treated with *PyQ*, we noticed that the CD45 distribution was modified, with an elevated number of CD45-clustered cells observed after treatment (Figure 5A). CD45 mostly localized within lipid rafts was no longer detected after *PyQ* treatment (Figure 5B). *PyQ* rapidly increased phosphorylation of Lyn at the inhibitory Y507 site, which is under the control of CD45 [25], while other signaling pathways (Pi3k, Akt, Erk) were not affected (Figure 5C). CD45 is well known in leukemia to be involved in the regulation of the GM-CSF pathway and we observed that *PyQ* treatment decreased the S727 phosphorylation of the Stat3 transcription factor (Figure 5D), and its target genes involved in growth and proliferation were consequently found inactivated (Figure 5E). *PyQ* is accordingly a potent inhibitor of GM-CSF signaling and this was confirmed by the administration of high dose of the recombinant GM-CSF, which antagonized significantly the inhibitory effect mediated by *PyQ* (Figure 5F and 5G). We furthermore confirmed this mechanism on the human hematopoietic THP1 line, with an elevated number of CD45-clustered cells observed after treatment with *PyQ* (Supplementary Figure S8A), delocalization of the CD45 phosphatase outside the lipid rafts (Supplementary Figure S8B), which increased phosphorylation of Lyn at its inhibitory site and inactivated the Stat3 pathway (Supplementary Figure S8C).

### CD45 colocalizes within lipid rafts on the cell surface of human AML blasts, but not on healthy HSCs

**Figure 6:**
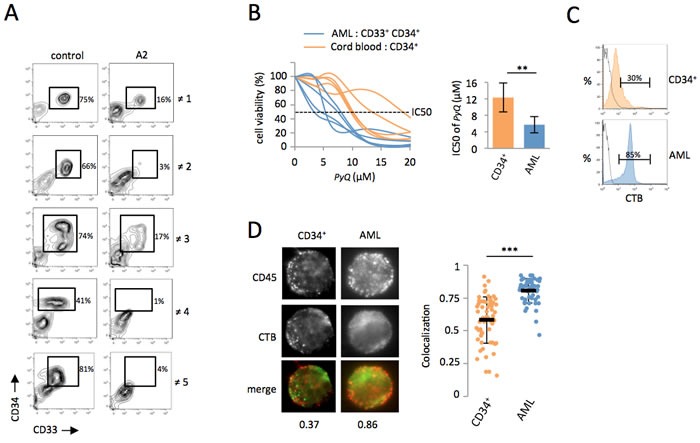
Primary human AML cells are sensitive to *Pyrido [4,3-b]quinoxaline* **A.** Total WBC were isolated by Ficoll from peripheral blood of five AML patients and treated *in vitro* with *PyQ* for 3 days. The percentage of blast cells (CD34^+^ CD33^+^) was shown after a treatment with *PyQ* (A2 at 6.8μM) or without (control). ≠1, AML3; ≠2, AML1; ≠3, AML2; ≠4, AML1; ≠5, AML4; data gated on viable cells. **B.** AML CD34^+^ CD33^+^ cells were more sensitive to *PyQ* treatment than normal primitive hematopoietic CD34^+^ cells purified from cord blood (*n* = 5 biological replicates). Viability was measured 3 days after treatment with various concentrations of *PyQ*, IC50 dose (right panel). **C.** CD34^+^ AML blasts were enriched in lipid rafts compared with CD34^+^ cord blood cells, as assessed by staining with CTB, measured by flow cytometry. Staining with isotype control antibody IgG was shown. **D.** CD34^+^ CD33^+^ leukemic blasts and CD34^+^ cord blood cells were sorted and analyzed by microscopy. Data showing that CD45 colocalized within lipid rafts (CTB) on CD33^+^ CD34^+^ AML blasts, but not on CD34^+^ cord blood cells. Examples of single cell immunostaining were shown on the left panel. Colocalization scores between CD45 and CTB were reported bellow the immunostaining. Colocalization means were calculated (*n* > 30 cells) and represented on the right panel. Mean ± SEM. **, *P* < 0.01; ***, *P* < 0.001; measured by Student's unpaired *t* test.

We next wanted to address if the positioning of the CD45 phosphatase within lipid rafts was also modified during the oncogenic transformation of human hematopoietic cells to acute myeloid leukemia. From peripheral blood of patients suffering of AML, we isolated leukemic blast that were *in vitro* treated with PyQ for 3 days. While CD34^+^ CD33^+^ leukemic myeloid blasts disappeared after a treatment with *PyQ*, the non-leukemic cells (CD33^−^), which principally contained lymphoid cells were found to be less affected (Figure 6A), suggesting that leukemic blasts were more sensitive to *PyQ* than healthy lymphoid cells. CD34^+^ CD33^+^ leukemic myeloid blasts were also more sensitive to *PyQ* than were healthy CD34^+^ HSCs isolated from cord blood (Figure 6B). We discovered that leukemic human myeloid blasts also contained a high level of lipid rafts, compared with the non-transformed human CD34^+^ HSCs (Figure 6C). Finally, we demonstrated that CD45 was differently organized on the cell surface, since CD45 colocalized more within lipid rafts on leukemia blasts than was the case in CD34^+^ cells (Figure 6D).

### Delocalization of CD45 outside lipid rafts affects the GM-CSF-dependent expansion of human AML cells

**Figure 7:**
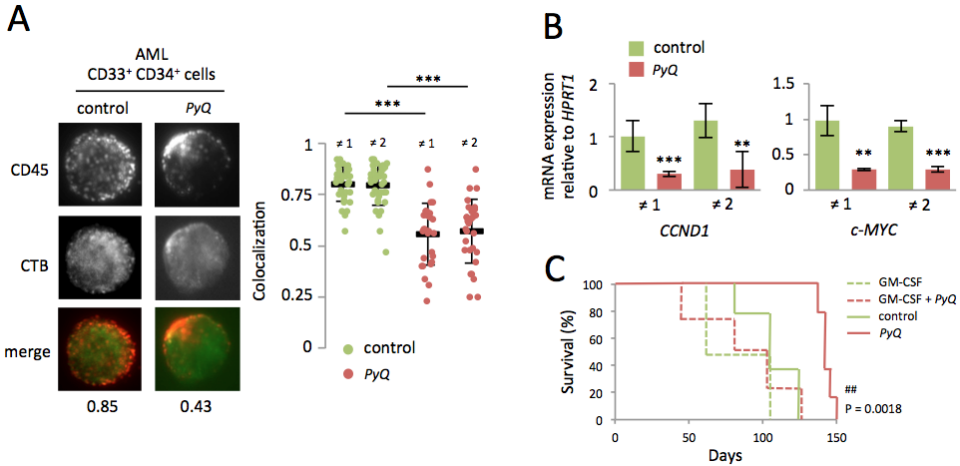
Localization of the CD45 within lipid rafts is important for maintenance of human AML cells **A.** After treatment of primary AML blasts with *PyQ* (6.8μM), CD45 was found delocalized outside the lipid rafts. Examples of single cell immunostaining were shown on the left panel (sample ≠1). Colocalization scores between CD45 and CTB were reported bellow the immunostaining. Colocalization means were calculated (*n* > 20 cells) and represented on the right panel for samples ≠1 and ≠2, under untreated (control) or treated (*PyQ*) conditions. **B.** Two hours after treatment with *PyQ*, Stat3 target genes were found down regulated on human CD34^+^ CD33^+^ sorted blast cells. **C.** Human AML leukemic cells were transplanted into lethally irradiated immunodeficient mice. Ten and thirteen days post-transplant, mice were given a tail injection of *PyQ* (3mg/Kg) alone or associated with high dose of the recombinant human GM-CSF (500ng/mouse). Kaplan-Meier survival curves of mice treated with *PyQ*, compared with control groups (*n* = 4 mice for GM-CSF and GM-CSF + *PyQ*, *n* = 5 mice for control and *PyQ*). Mean ± SEM. **, *P* < 0.01; ***, *P* < 0.001; measured by Student's unpaired *t* test. ^##^, *P* < 0.01; measured by the Mantel Haenszel logrank test, compared with control group.

CD45, which is mainly found within plasma-membrane lipid rafts on AML patients' blast cells, is rapidly delocalized after treatment with *PyQ* (Figure 7A). GM-CSF-Rα is expressed in more than 80% of human AML blasts [1-3], and GM-CSF is known to induce cell cycle progression [4, 5, 7, 8]. When cells were treated with *PyQ*, we observed a marked downregulation of GM-CSF/Stat3 target genes for human AML blasts (Figure 7B). Next, we tested the effect of *PyQ* on a xenograft model to evaluate its ability to block the development of leukemia. We transplanted fresh AML cells into the tail vein of lethally irradiated immunodeficient mice. Survival analyses showed that mice treated with *PyQ* survived significantly longer than untreated control mice (Figure 7C). Interestingly, we also provided evidence that administration of a supra-physiological dose of GM-CSF significantly blocked the efficacy of *PyQ*. In conclusion, we have shown that *PyQ* inactivates the CD45/GM-CSF pathway on human AML cells and could be used as a novel therapeutic agent for the treatment of AML.

## DISCUSSION

Lipid rafts are cholesterol- and glycosphingolipid-enriched patches located in the plasma membrane. They are a key component of the signal transduction pathway and contribute to signal intensity modulation of normal hematopoiesis. Lipid rafts are master regulators of cytokine function, the cell cycle, survival and the retention of HSCs [21-23]. Lipid rafts play also important roles in malignant hematopoiesis. The presence of CD45 within lipid rafts is responsible for IL-6 induced proliferation in myeloma [27, 28]. Aberrant JAK2 signaling in myeloproliferative neoplasms has been also described to be dependent on lipid rafts [29]. The therapeutic effect of *Rituximab* (anti-CD20) on B-cell lymphomas may depend on membrane cholesterol content, which promotes lipid raft formation [30]. Accumulating evidence suggests that lipid rafts also play important roles in AML, since physiological hypoxia induces lipid raft formation and PI3K activation in AML [31]. The *fluoropyrimidine F10*, which displays anti-leukemia activity in pre-clinical models of AML, reduces overall lipid raft levels in the plasma membrane [32]. The induction of apoptosis in AML cells by CD44 ligation with A3D8 antibody requires the presence of CD44 on lipid rafts [33].

CD45, a member of the protein tyrosine phosphatase (PTP) family specifically expressed in hematopoietic cells, is associated with lipid raft microdomains, and though the dynamic regulation of CD45 inside or outside lipid rafts has been well documented for lymphocyte activation in the immune response [14], its significance in leukemia is still unclear. We showed that the localization of CD45 on the cell surface of hematopoietic cells changed dynamically during their oncogenic transformation. On non-transformed mouse and human primitive hematopoietic cells, CD45 was observed outside lipid rafts, but once those cells had been transformed, CD45 was found within lipid rafts on AML blasts. By providing clear and detailed evidence, our study demonstrates that lipid rafts play a crucial role in both murine and human AML maintenance. Finally, these findings may lead to the development of new drugs, including *Pyrido [4,3-b]quinoxaline* (*PyQ*) for the treatment of hematological malignancies, *via* the pharmacological modulation of CD45 positioning among lipid rafts.

Genetic defects of CD45 in mice [34] and humans [35] cause severe combined immunodeficiency while no perturbation of myelopoiesis is observed, which demonstrates the essential role of CD45 in the immune system, especially for the activation and development of lymphocytes. Using CD45 deficient mice, we clearly showed the crucial role played by CD45 in the leukemogenic transformation process since mice transplanted with CD45KO cells never developed AML. It has been reported that CD45 regulates homing and engraftment of normal and leukemic cells [20, 36]. In our study, using lentiviral shRNA on HOXA9-MEIS1 blasts to knock down CD45, we observed that these resulting cells had lost their capacity to induce leukemia in secondary transplantation settings, even after inoculation of the cells into bone marrow, thus highlighting the pivotal role of CD45 in the maintenance of leukemia *in vivo*. Various studies suggest that AML stem cells arise from myeloid progenitors that adopt self-renewal properties. GM-CSF-Rα is certainly expressed in this myeloid progenitor sub-population in mice [37], but the biological significance of this receptor in the leukemic stem cell population is not yet well established. Here, we demonstrating that among the population of HOXA9-MEIS1 blasts, cells harboring a high level of CD45 (CD45^hi^) displayed enhanced activation of the GM-CSF/Stat3 pathway. Following transplantation of CD45^hi^ cells into secondary recipient mice, we discovered that, to a large extent, these cells were more leukemogenic. We can therefore conclude that the CD45/GM-CSF pathway plays a more critical role in the regulation of cancer stem cells than was previously thought.

In the past 20 years, numerous strategies have been employed to inhibit either the GM-CSF receptor [9, 10, 38, 39] or the downstream signaling, which includes Lyn kinase or Stat transcription factors [11, 12]. In this study, we revealed that *Pyrido [4,3-b]quinoxaline* can perturb the plasma membrane, and disrupts the dynamic positioning of CD45 within lipid raft microdomains. Using mouse and human AML samples, we demonstrated that this delocalization of CD45 inhibits the GM-CSF pathway, which is essential for the growth and survival of leukemic cells. We therefore provide new evidence that lipid rafts play a pivotal role in AML maintenance, and that the pharmacological modulation of lipid raft functions may lead to the development of new drugs for the treatment of leukemia.

## MATERIALS AND METHODS

### Mice

The Ethics Committee for Animal Welfare of the University of Bourgogne and the French Ministry of Higher Education and Research approved all animal experiments (references 01333.02 and 01318.02). Detailed protocol for animal studies and transplantation is provided in Supplemental experimental procedure. Human HOXA9 and MEIS1 (MSCV-IRES-GFP) retroviral vectors were transfected into Phoenix Eco cells and supernatants were harvested for infection of magnetically lineage-depleted (Lin^−^) cells isolated from bone marrow (BM) of C57BL/6 Wild type or CD45KO mice (depletion using a kit from Miltenyi). CD45KO Exon 9 mice were obtained from Pauline Johnson's laboratory (University of British Columbia, Vancouver, Canada). The CD45KO mice [34] were backcrossed for nine generations onto the C57BL/6 background. Transduced cells were transplanted into the tail vein of lethally irradiated (900cGy) in 7-12-wk-old C57BL/6 females. To test *PyQ* effect *in vivo* 5×10^4^ GFP^+^ leukemic cells were transplanted together with 5×10^4^ Lin^−^ cells isolated from B6SJL congenic (Ly.1) mice and 1×10^5^ cells from support total BM (Ly.2). *PyQ* (∼3mg/Kg) was injected into the peritoneum, 10 and 13 days post-transplantation. 5×10^4^ CD45^hi^ or CD45^lo^ cells were mixed with 1×10^5^ support BM cells and injected into the femur of lethally irradiated recipients. CD45 shRNA lentiviral particles (sc-35001-V, Santa Cruz Biotechnology, Inc.) were used to knock down CD45, after 18h of treatment with puromycin, 5×10^4^ cells were injected in BM.

### Human samples

Fresh cord blood samples were obtained from the Etablissement Français du Sang (Besançon, France). After Ficoll separation, CD34^+^ cells were isolated using the CD34 MicroBead Kit (Miltenyi). Patients were included, after giving their informed consent, in accordance with the Declaration of Helsinki. The present study was approved by the Local Ethic Committee of Dijon hospital. Detailed protocol for human studies is provided in Supplemental experimental procedure. AML diagnosis was based on the World Health Organization (WHO) criteria. Following Ficoll separation, white blood cells (WBC) were treated 3 days with *PyQ*. Kept frozen by the Biological Resource Center Ferdinand Cabanne at Dijon (BB-0033-00044), samples were analyzed for further investigations. AML sample was transplanted into the tail vein of lethally irradiated (300cGy) immunodeficient mice (NSG). *PyQ* (∼3mg/Kg) was injected into the tail vein, 10 and 13 days post-transplantation. Recombinant human GM-CSF (Miltenyi) was injected at 500ng per mouse.

### Chemical library

More than 7,400 chemical compounds from the French library (UMR176 CNRS, Institut Curie, Paris, France, http://curie.fr/recherche/plateforme-chimiotheque) were tested at 10ng/mL. Toxicities on HOXA9-MEIS1 leukemic and Lin^−^ cells were evaluated, by GFP^+^ and Propidium Iodide quantifications (BD Biosciences) respectively, using a 96-well-plate cytometer 18 hours after treatment (Guava EasyCyte PLUS, Millipore). The *PyQ* used in this study was A2.

### Flow cytometry and sorting

We followed previously described protocol [40]. Viability of HOXA9-MEIS1 AML cells was analyzed by GFP quantification using flow cytometry. Apoptosis was measured using an Annexin-V detection kit (BD Biosciences). Cell viability was analyzed using DAPI or Propidium Iodide (BD Biosciences). The effect of *Pyrido [4,3-b]quinoxalines* on the cell cycle was assessed by flow cytometry with anti-BrdU antibody, using the cell proliferation kit (BD Biosciences). AF488- and AF647-conjugated Cholera Toxin subunit B (Life Technologies) was used to stain lipid rafts. For immunostaining, we purified HSCs (LSK-CD34^−^) and the different progenitors (CMP, GMP) from Lin^−^ cells, following previously described protocol [40]. To analyze effect of *PyQ* on stem cells *in vivo*, (SLAM population) and progenitors (ST, MPP, CLP, CMP, GMP, MEP), we followed previously described protocol [40]. For murine cells, we used CD45.1-PE-Cy5.5 (110712, BioLegend), Stat3 (pS727)-AF647 (558099, BD Biosciences), CD45-APC-Cy7 (109824, BioLegend), CD45-APC-Cy7 (560694, BD Biosciences). For human cells, we used CD45-APC-Cy7 (557833, BD Biosciences), CD45-APC (555485, BD Biosciences), CD33-PE-Cy5.5 (B36289, Beckman Coulter), CD34-FITC (130-081-001, Miltenyi Biotech), CD34-APC (130-098-139, Miltenyi Biotech), Stat3 (pS727)-AF647 (558099, BD Biosciences). Cells were analyzed on a FACS LSRII and Canto10 flow cytometer (BD Biosciences) and sorted on a FACS Aria cell sorter (BD Biosciences). Data were analyzed using FlowJo software (Tree Star).

### *In vitro* culture

HOXA9-MEIS1 cells and human AML samples were maintained in SFEM (StemCell Technologies). Recombinant murine GM-CSF (Miltenyi) was tested at 25ng/mL. HOXA9-MEIS1 cells were grown on MS-5 stromal feeder cells, the co-culture was treated with *PyQ* for 3 days, trypsinized and feeder cells were isolated from leukemic cells by binding on 6-well plates. The recombinant human GM-CSF (Miltenyi) was tested on THP1 cells. Murine recombinant GM-CSF (Miltenyi) was used on HOXA9-MEIS1 cells. Recombinant murine GM-CSF and other murine cytokines (all from Miltenyi) were tested at 25ng/mL and administered 30min before the treatment with *PyQ*. CFU assay was performed on MethoCult medium (MethoCult, M3434).

### Cell lines

Hematopoietic and non-hematopoietic cell lines were reciprocally cultured in RPMI 1640 and DMEM (Pan Biotech), supplemented with 20% fetal bovine serum (Pan Biotech). Cell viability was analyzed using the XTT Cell Proliferation Kit (Roche Applied Science), four days after treatment with *PyQ*. CD45 shRNA lentiviral particles (sc-29251-V) and control particles (sc-108080) (Santa Cruz Biotechnology, Inc.) were used to knock down the CD45 on THP1.

### Immunostaining

Human and murine AML, CD34^+^ cord blood and Lin^−^ cells were stained with AF488-conjugated Cholera Toxin subunit B (Life technologies), anti-mouse or anti-human CD45 (clone F10-89-4, Millipore) and secondary anti-mouse-Alexa-Fluor-568, under non-permeabilized condition. Immunofluorescence was analyzed by microscopy (ZEISS, Imager.M2) and images were processed for colocalization studies (Fiji, NIH software).

### qPCR

Experiments were carried out using the Viia7 system (Applied Biosystems), with the following TaqMan assays. For mouse; Mm00432359 (Ccnd1), Mm00487425 (c-Fos), Mm00487804 (c-Myc), Mm04243546 (Jun-B), Mm01545399 (Hprt1), Mm01290062 (GM-CSF). For human; Hs00765553 (CCND1), Hs00153408 (c-MYC), Hs02800695 (HPRT1).

### Microarrays

The GSE57083 study provided normalized gene expression data for various human cell lines. Data were extracted for either hematopoietic lines NB4, HL60, THP1, KG1 and U937 or non-hematopoietic lines HeLa, SW480, HT29 and MCF7, and the study was focused on transcripts encoding proteins found in the “plasma membrane” and associated with “receptor activity”, which represent more than 170 mRNA.

### Western blotting

The antibodies used were anti-phospho-Stat3 (S727), anti-Stat3, anti-phospho-PI3K, anti-PI3K, anti-phospho-Erk (Y204), anti-Erk, anti-phospho-Lyn (Y507), anti-Lyn, anti-phospho-Akt (S473), anti-Akt (all from Cell Signaling Technology) and anti-Actin (BD Biosciences). Gel images were analyzed with ImageJ (NIH) for quantification.

### HPLC *chromatography*

*PyQ* was quantified in different subcellular fractions of THP1 cells by using high performance liquid chromatography (HPLC). To separate the cytoplasm from the nucleus, THP1 cells were reconstituted for 1min in cold buffer containing 0.5% NP-40, 10mM NaCl, 3mM MgCl_2_, 10mM Tris-HCl, pH 7.4. The cell lysis was then centrifuged for 1min at 500g. The pellet contains the nuclei, while the supernatant contains cytoplasm and plasma membranes. The supernatant was diluted twice with a buffer containing 150mM NaCl, 1mM EDTA, 1% Nonidet P-40, 50mM Tris-HCl, pH 8.0. After high-speed centrifugation (15,000g) for 15min, the pellet was enriched with plasma membrane fragments, while the supernatant contained the cytoplasm. DNA was precipitated with 100% ethanol, after treatment of the THP1 cells with *PyQ*.

### Statistics

All data are expressed as the mean ± SEM. Differences between two groups were assessed by Student's unpaired *t* test. Statistical analysis of survival curves was performed using the Mantel Haenszel logrank test. Statistics were performed using Prism 4 (GraphPad).

## SUPPLEMENTARY MATERIALS FIGURES



## References

[1] Budel LM, Touw IP, Delwel R, Clark SC, Lowenberg B (1989). Interleukin-3 and granulocyte-monocyte colony-stimulating factor receptors on human acute myelocytic leukemia cells and relationship to the proliferative response. Blood.

[2] Lanza F, Castagnari B, Rigolin G, Moretti S, Latorraca A, Ferrari L, Bardi A, Castoldi G (1997). Flow cytometry measurement of GM-CSF receptors in acute leukemic blasts, and normal hemopoietic cells. Leukemia.

[3] Park LS, Waldron PE, Friend D, Sassenfeld HM, Price V, Anderson D, Cosman D, Andrews RG, Bernstein ID, Urdal DL (1989). Interleukin-3, GM-CSF, and G-CSF receptor expression on cell lines and primary leukemia cells: receptor heterogeneity and relationship to growth factor responsiveness. Blood.

[4] Guan Y, Gerhard B, Hogge DE (2003). Detection, isolation, and stimulation of quiescent primitive leukemic progenitor cells from patients with acute myeloid leukemia (AML). Blood.

[5] Riccioni R, Diverio D, Riti V, Buffolino S, Mariani G, Boe A, Cedrone M, Ottone T, Foa R, Testa U (2009). Interleukin (IL)-3/granulocyte macrophage-colony stimulating factor/IL-5 receptor alpha and beta chains are preferentially expressed in acute myeloid leukaemias with mutated FMS-related tyrosine kinase 3 receptor. British journal of haematology.

[6] Guthridge MA, Barry EF, Felquer FA, McClure BJ, Stomski FC, Ramshaw H, Lopez AF (2004). The phosphoserine-585-dependent pathway of the GM-CSF/IL-3/IL-5 receptors mediates hematopoietic cell survival through activation of NF-kappaB and induction of bcl-2. Blood.

[7] Faderl S, Harris D, Van Q, Kantarjian HM, Talpaz M, Estrov Z (2003). Granulocyte-macrophage colony-stimulating factor (GM-CSF) induces antiapoptotic and proapoptotic signals in acute myeloid leukemia. Blood.

[8] Coffer PJ, Koenderman L, de Groot RP (2000). The role of STATs in myeloid differentiation and leukemia. Oncogene.

[9] Jakupovic I, Grandage VL, Linch DC, Khwaja A (2004). Lack of effect of the human GM-CSF analog E21R on the survival of primary human acute myeloid leukemia cells. Blood.

[10] Mathew M, Zaineb KC, Verma RS (2013). GM-CSF-DFF40: a novel humanized immunotoxin induces apoptosis in acute myeloid leukemia cells. Apoptosis.

[11] Hibbs ML, Harder KW (2006). The duplicitous nature of the Lyn tyrosine kinase in growth factor signaling. Growth Factors.

[12] Scapini P, Pereira S, Zhang H, Lowell CA (2009). Multiple roles of Lyn kinase in myeloid cell signaling and function. Immunol Rev.

[13] Wei S, Liu JH, Epling-Burnette PK, Gamero AM, Ussery D, Pearson EW, Elkabani ME, Diaz JI, Djeu JY (1996). Critical role of Lyn kinase in inhibition of neutrophil apoptosis by granulocyte-macrophage colony-stimulating factor. J Immunol.

[14] Mustelin T, Vang T, Bottini N (2005). Protein tyrosine phosphatases and the immune response. Nat Rev Immunol.

[15] Hermiston ML, Xu Z, Majeti R, Weiss A (2002). Reciprocal regulation of lymphocyte activation by tyrosine kinases and phosphatases. J Clin Invest.

[16] Simons K, Toomre D (2000). Lipid rafts and signal transduction. Nat Rev Mol Cell Biol.

[17] Golub TR, Slonim DK, Tamayo P, Huard C, Gaasenbeek M, Mesirov JP, Coller H, Loh ML, Downing JR, Caligiuri MA, Bloomfield CD, Lander ES (1999). Molecular classification of cancer: class discovery and class prediction by gene expression monitoring. Science.

[18] Kroon E, Krosl J, Thorsteinsdottir U, Baban S, Buchberg AM, Sauvageau G (1998). Hoxa9 transforms primary bone marrow cells through specific collaboration with Meis1a but not Pbx1b. EMBO J.

[19] Quere R, Andradottir S, Brun AC, Zubarev RA, Karlsson G, Olsson K, Magnusson M, Cammenga J, Karlsson S (2011). High levels of the adhesion molecule CD44 on leukemic cells generate acute myeloid leukemia relapse after withdrawal of the initial transforming event. Leukemia.

[20] Shivtiel S, Lapid K, Kalchenko V, Avigdor A, Goichberg P, Kalinkovich A, Nagler A, Kollet O, Lapidot T (2011). CD45 regulates homing and engraftment of immature normal and leukemic human cells in transplanted immunodeficient mice. Exp Hematol.

[21] Ratajczak MZ, Adamiak M (2015). Membrane lipid rafts, master regulators of hematopoietic stem cell retention in bone marrow and their trafficking. Leukemia.

[22] Yamazaki S, Iwama A, Takayanagi S, Morita Y, Eto K, Ema H, Nakauchi H (2006). Cytokine signals modulated *via* lipid rafts mimic niche signals and induce hibernation in hematopoietic stem cells. EMBO J.

[23] Jahn T, Leifheit E, Gooch S, Sindhu S, Weinberg K (2007). Lipid rafts are required for Kit survival and proliferation signals. Blood.

[24] Young RM, Holowka D, Baird B (2003). A lipid raft environment enhances Lyn kinase activity by protecting the active site tyrosine from dephosphorylation. J Biol Chem.

[25] Thomas ML, Brown EJ (1999). Positive and negative regulation of Src-family membrane kinases by CD45. Immunol Today.

[26] Hercus TR, Broughton SE, Ekert PG, Ramshaw HS, Perugini M, Grimbaldeston M, Woodcock JM, Thomas D, Pitson S, Hughes T, D'Andrea RJ, Parker MW, Lopez AF (2012). The GM-CSF receptor family: mechanism of activation and implications for disease. Growth Factors.

[27] Li FJ, Tsuyama N, Ishikawa H, Obata M, Abroun S, Liu S, Otsuyama K, Zheng X, Ma Z, Maki Y, Kawano MM (2005). A rapid translocation of CD45RO but not CD45RA to lipid rafts in IL-6-induced proliferation in myeloma. Blood.

[28] Zheng X, Li AS, Zheng H, Zhao D, Guan D, Zou H (2015). Different associations of CD45 isoforms with STAT3, PKC and ERK regulate IL-6-induced proliferation in myeloma. PLoS One.

[29] Griner LN, McGraw KL, Johnson JO, List AF, Reuther GW (2013). JAK2-V617F-mediated signalling is dependent on lipid rafts and statins inhibit JAK2-V617F-dependent cell growth. British journal of haematology.

[30] Nozaki Y, Mitsumori T, Yamamoto T, Kawashima I, Shobu Y, Hamanaka S, Nakajima K, Komatsu N, Kirito K (2013). Rituximab activates Syk and AKT in CD20-positive B cell lymphoma cells dependent on cell membrane cholesterol levels. Exp Hematol.

[31] Fiegl M, Samudio I, Mnjoyan Z, Korchin B, Fritsche H, Andreeff M (2010). Physiological hypoxia promotes lipid raft and PI3K-dependent activation of MAPK 42/44 in leukemia cells. Leukemia.

[32] Gmeiner WH, Jennings-Gee J, Stuart CH, Pardee TS (2015). Thymineless death in F10-treated AML cells occurs *via* lipid raft depletion and Fas/FasL co-localization in the plasma membrane with activation of the extrinsic apoptotic pathway. Leuk Res.

[33] Qian H, Xia L, Ling P, Waxman S, Jing Y (2012). CD44 ligation with A3D8 antibody induces apoptosis in acute myeloid leukemia cells through binding to CD44s and clustering lipid rafts. Cancer Biol Ther.

[34] Byth KF, Conroy LA, Howlett S, Smith AJ, May J, Alexander DR, Holmes N (1996). CD45-null transgenic mice reveal a positive regulatory role for CD45 in early thymocyte development, in the selection of CD4+CD8+ thymocytes, and B cell maturation. J Exp Med.

[35] Kung C, Pingel JT, Heikinheimo M, Klemola T, Varkila K, Yoo LI, Vuopala K, Poyhonen M, Uhari M, Rogers M, Speck SH, Chatila T, Thomas ML (2000). Mutations in the tyrosine phosphatase CD45 gene in a child with severe combined immunodeficiency disease. Nat Med.

[36] Shivtiel S, Kollet O, Lapid K, Schajnovitz A, Goichberg P, Kalinkovich A, Shezen E, Tesio M, Netzer N, Petit I, Sharir A, Lapidot T (2008). CD45 regulates retention, motility, and numbers of hematopoietic progenitors, and affects osteoclast remodeling of metaphyseal trabecules. J Exp Med.

[37] Pronk CJ, Rossi DJ, Mansson R, Attema JL, Norddahl GL, Chan CK, Sigvardsson M, Weissman IL, Bryder D (2007). Elucidation of the phenotypic, functional, and molecular topography of a myeloerythroid progenitor cell hierarchy. Cell Stem Cell.

[38] Frankel AE, Powell BL, Hall PD, Case LD, Kreitman RJ (2002). Phase I trial of a novel diphtheria toxin/granulocyte macrophage colony-stimulating factor fusion protein (DT388GMCSF) for refractory or relapsed acute myeloid leukemia. Clin Cancer Res.

[39] Kreitman RJ, Pastan I (1997). Recombinant toxins containing human granulocyte-macrophage colony-stimulating factor and either pseudomonas exotoxin or diphtheria toxin kill gastrointestinal cancer and leukemia cells. Blood.

[40] Quere R, Saint-Paul L, Carmignac V, Martin RZ, Chretien ML, Largeot A, Hammann A, Pais de Barros JP, Bastie JN, Delva L (2014). Tif1gamma regulates the TGF-beta1 receptor and promotes physiological aging of hematopoietic stem cells. Proc Natl Acad Sci U S A.

