# Supplementary material for: CD45 phosphatase is crucial for human and murine acute myeloid leukemia maintenance through its localization in lipid rafts
Source: Oncotarget. 2016 Aug 25;7(40):64785–97. doi: 10.18632/oncotarget.11622 (PMC5323116; doi:10.18632/oncotarget.11622)
Supplement: Supplementary file 1 [file oncotarget-07-64785-s001.pdf]

CD45 phosphatase is crucial for human and murine acute myeloid leukemia maintenance through its localization in lipid rafts

Supplementary Material

Supplementary Experimental Procedure

Kinase assay. The International Centre for Kinase Profiling performed the kinase screening (University of Dundee, Scotland, UK).

Lipid analyses. Artificially double-layered membranes (micelles) were synthesized by adding *Lα-Phosphatidylcholine* (P5888, Sigma Aldrich) to water (1:1 v/v). *PyQ* A2 was then added to the mixture, vortexed and after centrifugation, *PyQ* A2 in the different fractions was quantified by HPLC.

Sucrose density gradient ultracentrifugation. Cells were washed with ice-cold 1xPBS and potted with 1 mL MES buffer (Sigma-Aldrich) containing 150mM NaCl on ice. In ultra-clear Tubes 14x89mm (Beckman Coulter), 2mL of lysates were mixed with the 2ml of 90% sucrose in lysis buffer to obtain 45% sucrose and overlaid with 5 mL of 35% sucrose in buffer followed by 3 mL of 5% sucrose in buffer. After ultracentrifugation for 20 hours at 39,000 rpm, 8 fractions were collected starting from the top of the gradient. Quantification of cholesterol and sphingomyelin in fraction was performed by the Lipidomic Analytical Platform (Université de Bourgogne, Dijon, France). Fractions were analyzed by WB using anti-CD45 (clone 69/CD45, BD Biosciences), anti-Lyn and anti-Flot1 antibodies (Cell Signaling Technology).

Supplementary References

Byth, K.F., Conroy, L.A., Howlett, S., Smith, A.J., May, J., Alexander, D.R., and Holmes, N. (1996). CD45-null transgenic mice reveal a positive regulatory role for CD45 in early thymocyte development, in the selection of CD4+CD8+ thymocytes, and B cell maturation. J Exp Med *183*, 1707-1718.
Quere, R., Saint-Paul, L., Carmignac, V., Martin, R.Z., Chretien, M.L., Largeot, A., Hammann, A., Pais de Barros, J.P., Bastie, J.N., and Delva, L. (2014). Tif1gamma regulates the TGF-beta1 receptor and promotes physiological aging of hematopoietic stem cells. Proc Natl Acad Sci U S A *111*, 10592-10597.

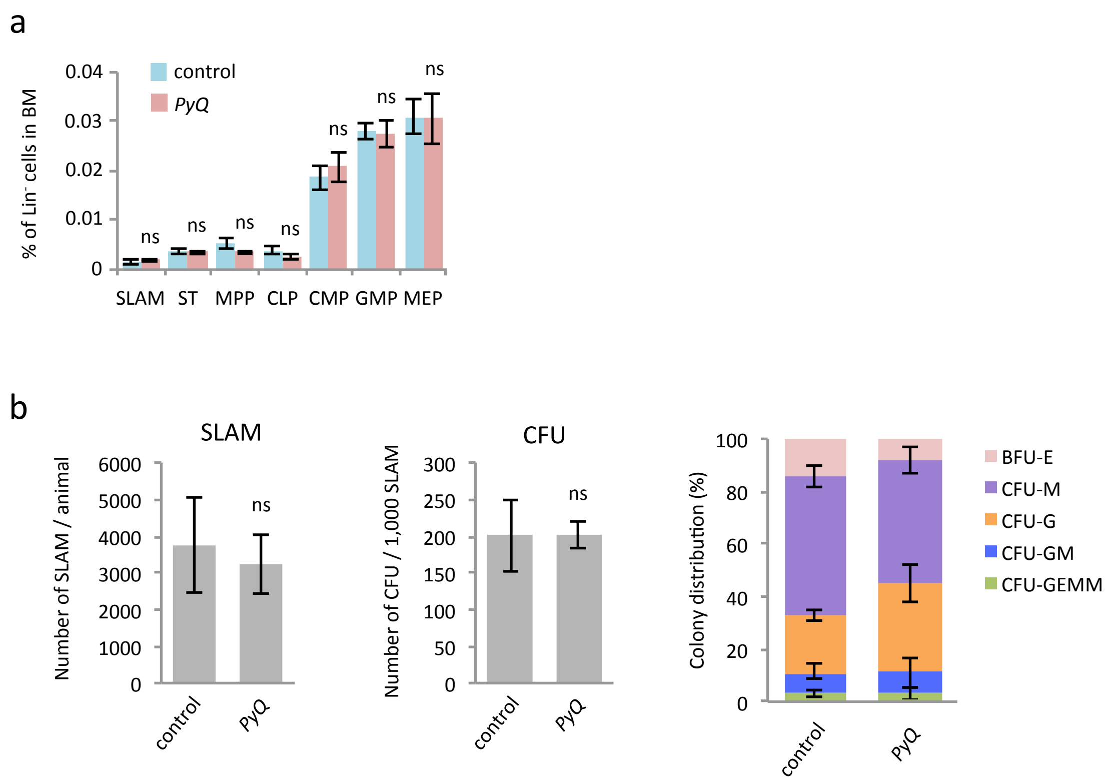

Figure S1. Toxicity of *Pyrido[4,3-b]quinoxaline* was evaluated on primitive hematopoietic stem/progenitor cells in bone marrow. (**a**) *PyQ* injected in mice did not have any effect on stem cells and progenitors distribution in bone marrow. (**b**) Primitive SLAM stem cells isolated from Lin$^-$ BM of mice treated with *PyQ* show the same potency to give rise to colony-forming unit (CFU) on MethoCult medium. Mean ± SEM. ns, non-significant, P value measured by Student's unpaired t test.

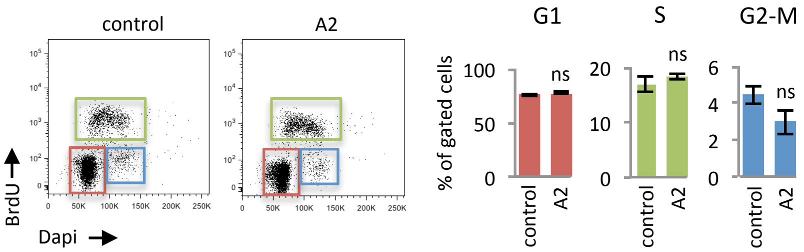

Figure S2. *PyQ* does not change cell cycle activity in HOXA9-MEIS1 cells. Cell cycle activity of HOXA9-MEIS1 cells exposed to 3.4μM *PyQ* for 6h. Data were gated on GFP^+ leukemic cells. Mean ± SEM. ns, non-significant, P value measured by Student's unpaired t test.

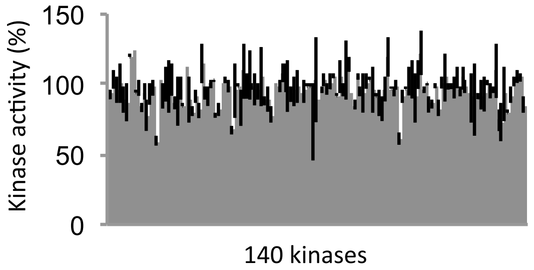

Figure S3. *Pyrido[4,3-b]quinoxaline* is not an inhibitor of kinases. Graphical representation of the kinase activity, n = 2 technical replicates, for 140 kinases tested with *PyQ* (3.4μM).

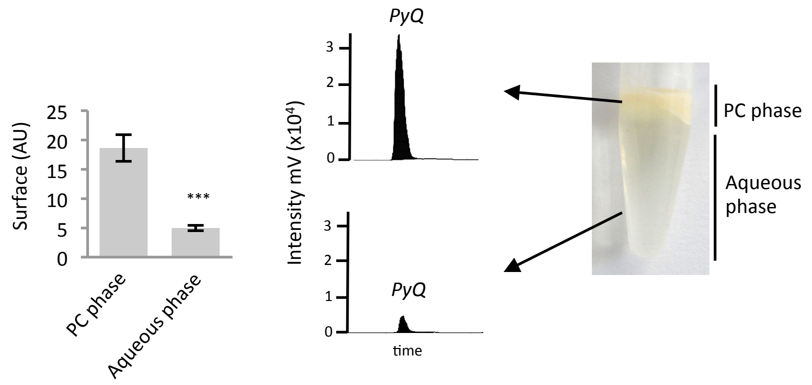

Figure S4. *PyQ* interacts with artificial lipid membranes (micelles) made by adding *Lα-phosphatidylcholine* (PC) to water. *PyQ* was quantified by HPLC chromatography, n = 3 technical replicates. Mean ± SEM. ***, P < 0.0001; measured by Student's unpaired t test.

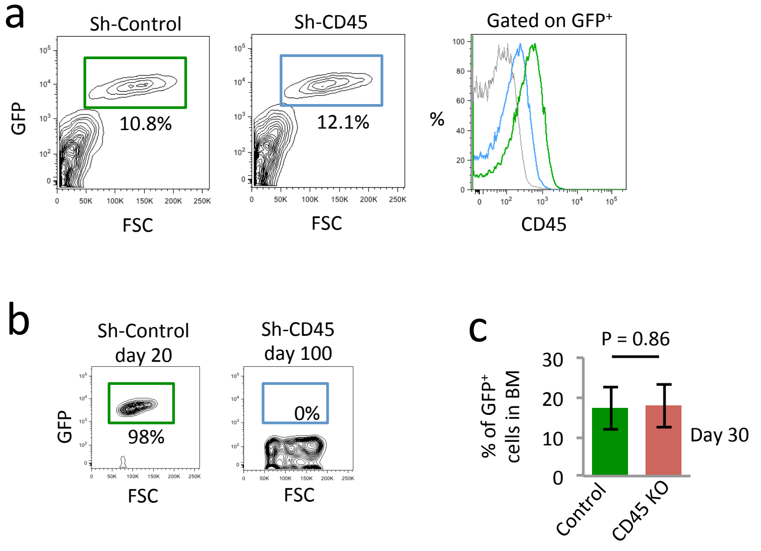

Figure S5. CD45 is critical for the maintenance and development of AML. (**a**) Using shRNA lentivirus, CD45 was knocked down in AML cells. CD45 down regulation was observed by cytometry on GFP^+ leukemic cells, after selection with puromycin. (**b**) Cells were then transplanted into lethally irradiated recipient mice. While mice from the Sh-Control group rapidly developed AML, 100 days after transplantation, surviving mice from the Sh-CD45 group were killed and no GFP^+ leukemic cells were detectable in BM by cytometry (related to Figure 2d). (**c**) Deficiency in CD45 expression completely prevented CD45 KO primitive hematopoietic cells from leukemic transformation. One month after the transplantation, we detected similar reconstitution in peripheral blood of Control- and CD45 KO-transplanted mice. However, deficiency in CD45 expression completely prevented CD45 KO primitive hematopoietic cells from leukemic transformation (related to Figure 2e). Control; n = 10 mice, CD45 KO; n = 10 mice. Mean ± SEM. P value measured by Student's unpaired t test.

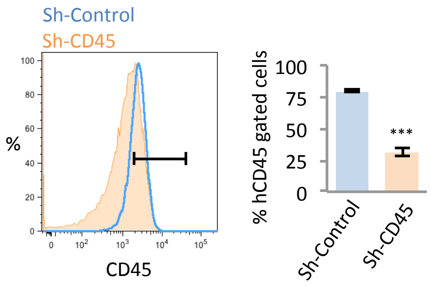

Figure S6. Knock down of CD45 observed by cytometry on THP1 leukemic cells, after selection with puromycin. THP1 cells were transduced with shRNA lentiviral particles (Sh-CD45 or Sh-Control). Mean ± SEM. ***, $P < 0.0001$; measured by Student's unpaired t test.

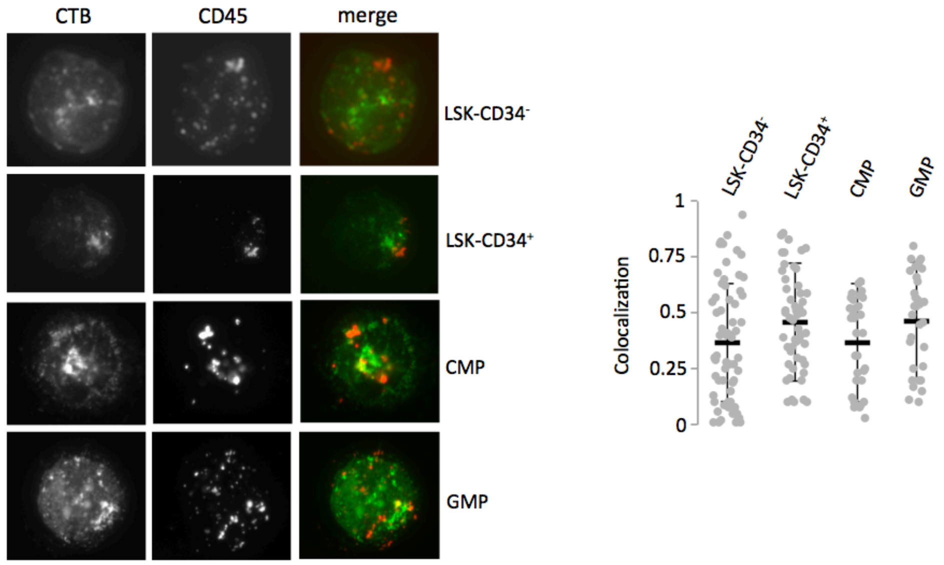

Figure S7. The CD45 was not colocalized within lipid rafts on different populations of hematopoietic stem and progenitor cells purified from Lin$^-$ cells. Lipid rafts were stained with the Cholera Toxin subunit B (CTB). The population of Lin$^-$ Sca1$^+$ c-Kit$^+$ (LSK) CD34$^-$ cells is enriched of HSCs. From the population of Lin$^-$ c-Kit$^+$ (LK), we isolated the common myeloid progenitors (CMP) and granulocyte monocyte progenitors (GMP). Examples of immunostaining are shown (left panel) and statistic ($n > 20$ cells, right panel).

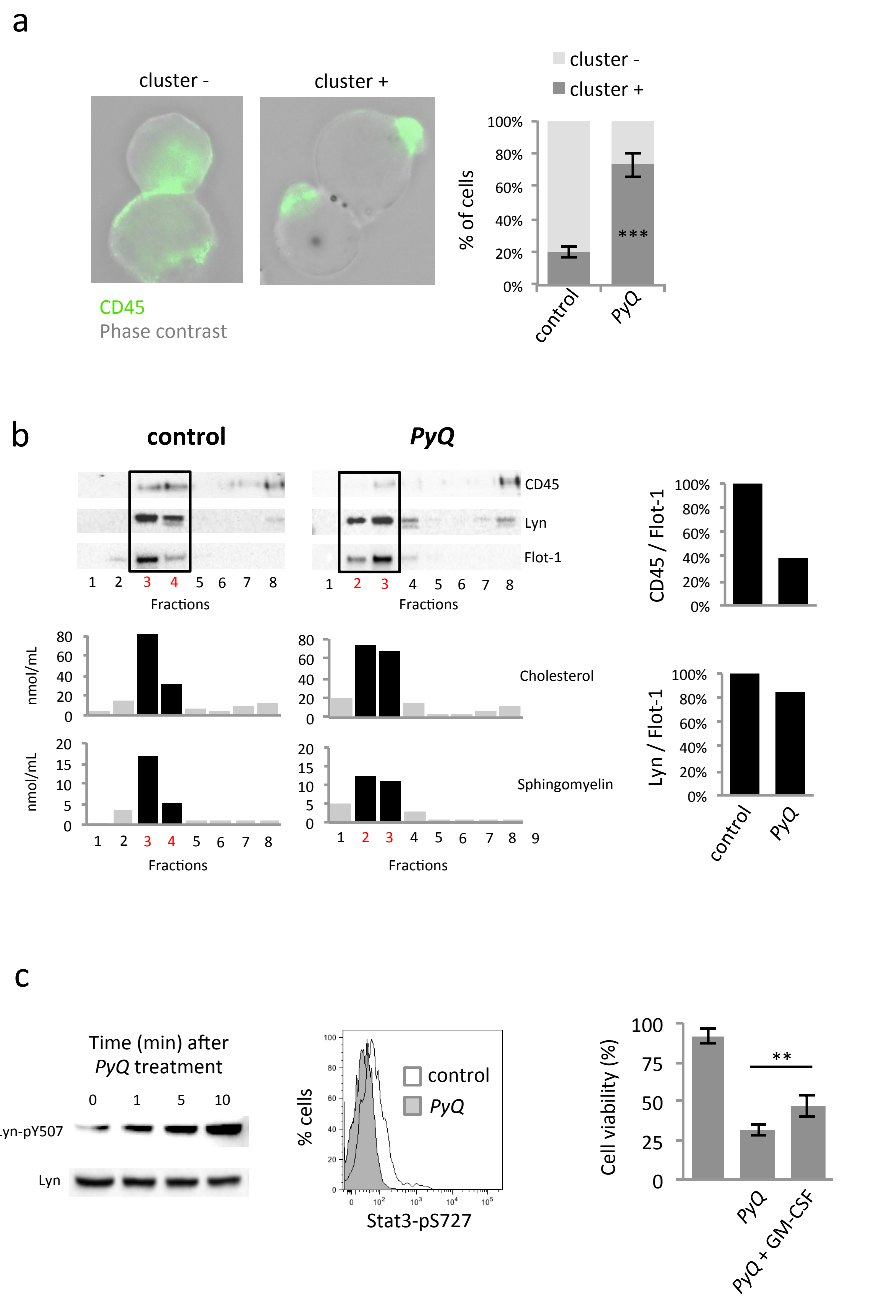

Figure S8. *Pyrido[4,3-b]quinoxaline* modifies the CD45 phosphatase localization and inactivates the Lyn/Stat3 pathway on THP1 cell line. (**a**) Quantification of the CD45 clusterisation induced by *PyQ* (13.6µM) for 15 min on THP1 cells. CD45 was either observed dispersed on the cell surface (cluster -) or clustered (cluster +). Examples of immunostaining are shown (magnification X63) and statistic (n > 20 cells). (**b**) When THP1 cells were treated with *PyQ* (13.6µM) for 15 min, CD45 was found delocalized outside from the lipid rafts. Lysates obtained from THP1 cells were separated by sucrose density gradient centrifugation. Lipid rafts were identified by WB with anti-Flot1 and by quantification of cholesterol and sphingomyelin via HPLC. On the right is shown the quantification of the WB demonstrating that CD45 was found delocalized outside from the lipid rafts after a treatment with *PyQ*, while Lyn remained inside. (**c**) THP1 cells were treated with *PyQ* (13.6µM) at different time ranging from 0 to 10 min. WB indicates phosphorylation of the negative regulatory site of Lyn (Y507) (left panel). After a treatment with *PyQ*, decreased phosphorylation of Stat3

(S727) was observed by flow cytometry, 40 min after A2 treatment (middle panel). Administration of recombinant GM-CSF (50ng/mL) in the culture medium reduced efficiency of *PyQ* (13.6μM) on THP1 cell viability after 3 days of treatment, n = 3 biological (right panel). Mean ± SEM. **, P < 0.001; ***, P < 0.0001; measured by Student's unpaired t test.
